# Editorial: B-cell deficiency and infection: exploring humoral immunity’s protective mechanisms

**DOI:** 10.3389/fimmu.2025.1678416

**Published:** 2025-08-28

**Authors:** Alexandros Grammatikos, Peter Bergman

**Affiliations:** ^1^ North Bristol National Health Service Trust, Bristol, United Kingdom; ^2^ Karolinska Institutet, Stockholm, Sweden

**Keywords:** B cell, infection, alymphocytosis, low B cells, immune deficiency, inborn error immunity

B-cells are vital components of our immune system, acting not only through antibody production but also as antigen-presenting cells and cytokine producers. Patients with disturbed B cell development and differentiation, such as those with agammaglobulinemia or Good’s syndrome, are notably susceptible to infections, including atypical and opportunistic pathogens. Recent studies have also highlighted their increased vulnerability and poorer outcomes when faced with infections like SARS-CoV-2, norovirus, enterovirus, and Campylobacter. Interestingly, this susceptibility seems to be independent of the often associated antibody deficiency but the underlying mechanisms remain unclear. This Research Topic aims to bridge the gap in our understanding of the pathogenic mechanisms that underlie infectious complications in these patients.

X-linked agammaglobulinemia (XLA) is the prototypical inborn error of immunity (IEI) in which patients present with very few or no B cells in peripheral blood. These patients are known to have a severe block in B cell maturation and poor antibody production due to mutations in Bruton’s tyrosine kinase (BTK) gene. del Pino-Molina et al. report a novel BTK variant detected in an apparently healthy 14-year-old male with an incidental finding of low IgM and reduced peripheral B-cell counts. Using an advanced flow cytometry assay and by analysing bone marrow samples they identified that, in contrast to classical XLA, only a minimal block in B-cell differentiation was observed in this patient. This finding offers a valuable insight into the role of BTK gene in B cell development and the spectrum of diseases associated with BTK mutations. ​

Another immunodeficiency in which peripheral B cells classically are very low or absent is Good’s Syndrome (GS). GS is characterized by the coexistence of thymoma and hypogammaglobulinemia, but the immunological characteristics in this disease are otherwise not well defined. With the use of highly sensitive flow cytometry Torres-Valle et al. identified that B-cells were reduced in all GS patients tested, with counts being significantly lower compared to patients with Common Variable Immune Deficiency. Interestingly, a subset of GS patients developed hypogammaglobulinemia several years after their thymoma was detected and these patients seemed to have a poorer response to immunoglobulin replacement therapy with a higher rate of opportunistic infections. Immunophenotyping in these patients identified reduced counts of central memory T cells, total T follicular helper cells, and total Th2 cells, as well, highlighting the complex interplay between B and T cells in their phenotypic manifestation.

LRBA haploinsufficiency is another IEI that is linked to recurrent infections. LRBA is an intracellular protein involved in receptor recycling and its deficiency is known to result in abnormal CTLA4 expression in T cells, and immune dysregulation. However, the exact pathogenic mechanisms that underlie the predisposition to infections in these patients, are not well understood. Flores-Hermenegildo et al. are shedding more light into the finding of higher IgA production that is seen in Lrba deficient mice. They identified that Lrba influences TGFbR recycling, with signalling through the latter known to be important for the differentiation of IgA positive B cells. Increased TGFbRII degradation likely explains the increased IgA production in these mice. This suggests that B cells may also play a role in the pathogenesis of this condition, although further work is required to elucidate their exact role and any potential association to infections.

Finally, Delgado et al. report on an automated database-guided gating and identification (AGI) approach for the use with multiparameter flow cytometry (FC) immunophenotyping. The currently used strategies for conventional data analysis are time consuming, require expertise, and show limited reproducibility. The authors use human B-lymphocyte and plasma cell populations to validate this approach. ​The AGI tool demonstrated a high degree of correlation with expert-based manual gating, a lower degree of variability, and a significantly reduced time requirement for data analysis compared to manual gating. This novel tool can have important applications in the characterization of immune cell alterations in both immunodeficiency and infections, making it an excellent candidate to study patients with B cell related diseases.

In summary, these four articles offer critical insights into the role of B cells in immunity to infection through the study of different IEIs associated with aberrant B cell function. They also highlight the importance of flow cytometry as a research and diagnostic tool to characterise patients with B cell alymphocytosis. Future studies are expected to inform the clinical decision-making on the use of B-cell-depleting therapies as well, and the potential use of circulating B-cell counts as a risk stratification marker.

